# Anticancer Potential of Tocopherols-Containing Plants and Semi-Synthetic Tocopherols

**DOI:** 10.3390/plants13212994

**Published:** 2024-10-26

**Authors:** Nesti Fronika Sianipar, Zidni Muflikhati, Dave Mangindaan, Khoirunnisa Assidqi

**Affiliations:** 1 Biotechnology Department, Faculty of Engineering, Bina Nusantara University, Jl. KH Syahdan No. 9, Jakarta 11480, Indonesia; khoirunnisa.assidqi@binus.edu; 2 Food Biotechnology Research Center, Bina Nusantara University, Jl. KH Syahdan No. 9, Jakarta 11480, Indonesia; zidnimufli44@gmail.com; 3 Waste-Food-Environmental Nexus Research Interest Group, Bina Nusantara University, Jl. KH Syahdan No. 9, Jakarta 11480, Indonesia; 4 Civil Engineering Department, Faculty of Engineering, Bina Nusantara University, Jl. KH Syahdan No. 9, Jakarta 11480, Indonesia; 5 Professional Engineering Program Department, Faculty of Engineering, Bina Nusantara University, Jl. KH Syahdan No. 9, Jakarta 11480, Indonesia

**Keywords:** medicinal plants, anticancer, bioactive compound, tocopherol, semi-synthetic compound, literature review

## Abstract

Tocopherols, potent bioactive compounds with anticancer properties, remain understudied in herbal medicinal plants, presenting a significant knowledge gap in the field of natural anticancer agents. This review evaluates tocopherol-containing plants for their anticancer potential, analyzing Scopus publications from 2016 to 2024. Fifteen herbal medicinal plants were identified as promising candidates, including *Bulbine anguistifolia* Poelln, *Punica granatum* L., *Moringa oleifera*, *Kigelia pinnata*, and *Typhonium flagelliforme* Lodd. The review explores tocopherols’ anticancer mechanisms, including apoptosis induction and cell cycle arrest. Factors influencing tocopherols’ anticancer effects are examined, such as their forms (α, β, γ, δ), concentrations, plant parts utilized, and their stability in various plants. Additionally, emerging research on semi-synthetic tocopherol derivatives is analyzed, highlighting their potential as adjuvants in chemotherapy and their role in enhancing drug delivery and reducing side effects. This comprehensive analysis aims to advance the development of plant-based anticancer pharmaceuticals and improve cancer treatment strategies. By elucidating the mechanisms and potential of tocopherol-containing plants, this review provides a foundation for future research in plant-based anticancer therapies. It emphasizes the need for further investigation into these plants’ anticancer properties, potentially leading to novel, more effective, and less toxic cancer therapies. The findings presented here contribute to a nuanced understanding of how tocopherol-containing plants can be leveraged in the development of future anticancer drugs.

## 1. Introduction

Bioactive compounds are substances with biological activity that directly affect living organisms. The effects of bioactive chemicals can differ based on the substance, dosage, and bioavailability [1]. However, bioactive compounds consumed at sufficient levels can provide medicinal advantages, including avoiding and curing several illnesses [2]. Recently, the increase in illnesses, including heart attack, obesity, and carcinoma, has led to the development of research on herbal plants as drugs in the pharmaceutical industry sector for cancer prevention. Bioactive compounds are found in fruits, vegetables, and grains [3,4]. They belong to a very heterogeneous class of compounds (polyphenolic compounds, carotenoids, phytosterols, and tocopherols) with different chemical structures (hydrophilic or lipophilic) [3,5].

Tocopherols are various forms of vitamin E, which is a group of bioactive, fat-soluble compounds composed of tocopherols and tocotrienols as two main classes, essential for human nutrition [6]. Alpha-tocopherol is the most biologically active among beta-, gamma- and delta-tocopherol in human. According to epidemiological studies, vitamin E could potentially reduce cancer and heart attacks, increase the immune system’s efficiency, and protect against various age-degenerative conditions (cataracts, spinal disorders, and arthritis) [7]. The antioxidant vitamin E supplements, have been used for its antioxidant qualities to reduce the risk of developing cancer [7]. Tocopherols have different variants, namely four tocopherol groups: α, β, γ, and δ. The difference in tocopherol groups lies in their function and ability to inhibit cells [8]. Tocopherol derivatives can be synthesized through various chemical modifications, which have been shown to enhance their anti-cancer properties, making them promising candidates for further pharmacological research [9]. Plants can be the main source of the body’s antioxidant vitamin E since tocopherols can be exclusively generated by photosynthesis in organisms like plants.

Exploring medicinal plants for anticancer agents is important as these plants have been used in traditional medicine for centuries and may offer novel compounds that could enhance cancer treatment [10]. Plants may produce a range of chemical compounds with biochemical and medicinal properties and are ideal for research as basic therapeutic ingredients, including anticancer [11]. The potential of plants being employed as therapeutic agents is partly because of their accumulation of bioactive chemicals [12]. Some herbal medicinal plants that can be used as inhibitors of cancer cell growth, are *Typhonium flagelliforme* [13,14], *Moringa oleifera* [15,16], *Zingiber officinale* [17,18,19], *Curcuma longa* [20,21], *Withania somnifera* [22,23], and *Pisum fulsum* [24,25].

In some herbal medicinal plants, tocopherol content is found in all plant parts, including leaves, tubers, stems, roots, and seeds [26]. Plants that have tocopherol content, as showcased in Table 1, include *Bulbine anguistifolia* Poelln (Asphodelaceae family), *Moringa oleifera* (Moringaceae family)*, Kigelia pinnata* (Bignoniaceae family), *Cissus assamica* (Vitaceae family), *Asclepias curassavica* L. (Apocynaceae family), and *Typhonium flagelliforme* (Araceae family) [27,28,29,30,31,32,33]. 

Despite the promising potential of tocopherols as an anticancer agent, research on tocopherol-containing plants has been relatively limited compared to other bioactive compounds such as polyphenols and carotenoids [34,35,36]. This research gap can be attributed to several factors, including the complexity of isolating and studying fat-soluble compounds like tocopherol, the historical focus on synthetic vitamin E in medical research, and challenges in standardizing tocopherol content across different plant sources. However, the unique properties of plant-based tocopherols, including their natural co-occurrence with other beneficial plant compounds and potential synergistic effects, make them worthy of increased research attention. Furthermore, the growing interest in natural product-based pharmaceuticals underscores the timely nature of focused research on tocopherol-containing plants for anticancer applications.

This review discusses herbal medicinal plants that contain potential anticancer tocopherols and may be developed as anticancer drugs in further research. When testing anticancer activity using unpurified extracts, it is important to consider that the observed effects may not be solely due to the compound present in the highest concentration. The interactions between various components in the extract, including potential synergistic or antagonistic effects, can significantly influence the overall bioactivity. This review selects medicinal plants from Scopus publications from 2016–2024 because Scopus provides a comprehensive collection of multidisciplinary research, making it suitable for our focus on medicinal plants and tocopherols. Scopus was chosen for its extensive coverage of peer-reviewed literature and its robust content curation process overseen by the Content Selection and Advisory Board [37,38,39]. It appears that reviews on herbal medicinal plants containing tocopherol for anticancer purpose are not available. Furthermore, it explores the emerging field of semi-synthetic tocopherol derivatives, potentially leading to improved therapeutic outcomes in cancer treatment. The objectives of this review are as follows: (1) To select publications containing tocopherols with anticancer activities. (2) To analyze the various forms and concentrations of tocopherols in the selected medicinal plants or extracts and their contribution to anticancer activities. (3) To identify factors influencing the anticancer activities of tocopherols in the selected plants, including common traits among species, plant parts utilized, tocopherol forms and concentrations, the presence of other phytochemicals, stability, degradation, and analytical methods employed.

## 2. Tocopherol Amounts in Selected Herbal Medicinal Plant Species

**Figure 1:**
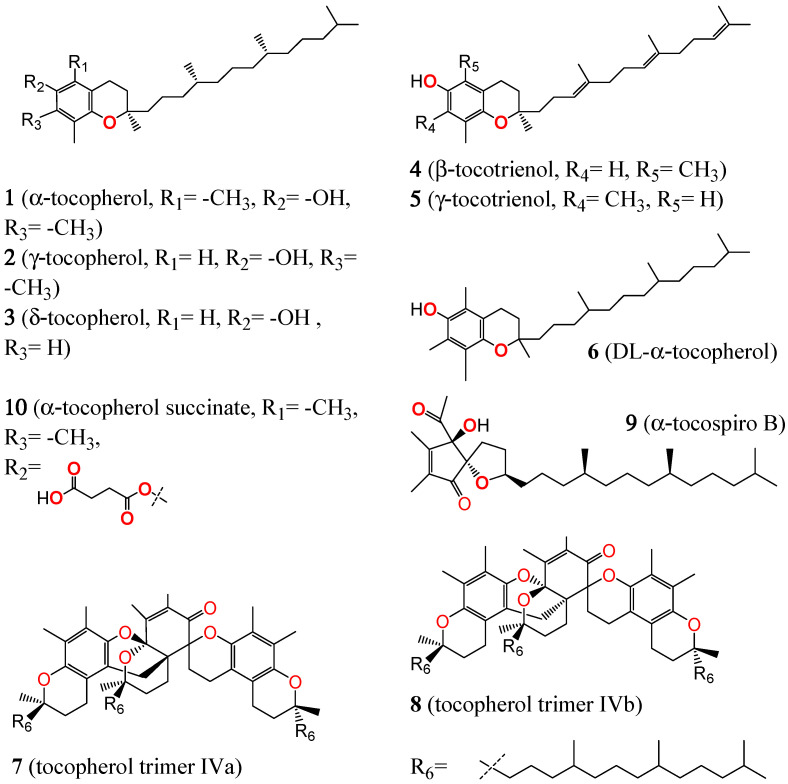
Chemical structures of tocopherols (**1**–**3**, **6**–**8**), tocotrienols (**4**–**5**), α-tocospiro B (**9**), and α-tocopherol succinate (**10**). Structure numbers correspond to the “Structure Number” column in Table 1. α-tocopherol (**1**), γ-tocopherol (**2**), δ- tocopherol (**3**), β-tocotrienol (**4**), γ-tocotrienol (**5**), DL-α-tocopherol (**6**), tocopherol trimer IVa (**7**) tocopherol trimer IVb (**8**), α-tocospiro B (**9**), and α-tocopherol succinate (**10**).

Anticancer tocopherol variants in herbal medicinal plants and their biological activity are shown in Table 1, with a total of 15 publications derived from the literature search (see Section 5 for literature search and plant selection method). Table 1 presents the results of diverse studies aimed at extracting tocopherol variants from various medicinal plant species, focusing on inhibiting cancer cells. The chemical structure forms of tocopherol compounds presented in Table 1 are illustrated in Figure 1. This figure showcases the structural variants of tocopherols, which are essential for understanding their biochemical properties and potential anticancer activities. By highlighting the different forms of tocopherols, this visual representation emphasizes the diversity of these bioactive compounds and their implications for medicinal applications.

**Table 1:** Compilation of existing studies on anticancer tocopherol variants in herbal medicinal plants.

No.	Plant Species	Plant Source	Biological Activity	Total Tocopherol Content	Structure Number	IC_50_ & Cancer Cell Lines	Reference
1.	*Bulbine anguistifolia* Poelln (Asphodelaceae family)	Roots extracts	Exhibit significant cytotoxic effects against cancer cells, indicating potential as an anticancer agent.	α-tocopherol succinate *: 4.16%	**10**	- IC_50_ of acetone extract = 28.21 μg/mL, COX-2 - LC_50_ of dichloromethane extract (50% lethal concentration) = 22.46 μg/mL - MCF-7 cells and Caco-2 cell line	[28]
2.	*Punica granatum* L. (Punicaceae family)	Fruits extracts	Red pomegranate exhibited the highest cytotoxic activity among the three varieties, followed closely by easy red and white pomegranates, which displayed comparable effects.	Total tocols (α-, γ-, δ-tocopherol, and β-, γ-tocotrienol): - 32.1 ± 0.2 μg (white pomegranate) - 69.4 ± 1.0 μg (pink pomegranate) - 110.0 ± 0.2 μg (red pomegranate)	α-tocopherol: **1** γ-tocopherol: **2** δ- tocopherol: **3** β-tocotrienol: **4** γ- tocotrienol: **5**	- IC_50_ > 200 μg/mL - MCF-7 cells (breast), HCT116 cells (colorectal), PC-3 cells (prostate), A549 cells (lung)	[40]
3.	*Calligonum comosum* (Polygonaceae family)	Leaves extracts	Significant anticancer for MCF-7 and MDA 231 cancerous breast cells. Has a specific apoptotic effect on cancer cells and shows minimal toxicity against non-cancerous breast cells (MCF-12).	DL-α-tocopherol: 14.75%	**6**	- IC_50_ = 269 μg/mL, MCF-7 cells - IC_50_ = 258 μg/mL - MDA 231 cells, MCF-7 cells	[41]
4.	*Moringa oleifera* (Moringaceae family)	Seed extract	Significant in vitro anticancer effects have been observed, demonstrating potent inhibitory activity against target proteins associated with breast and prostate cancer.	α-tocopherol: - 13.76 ± 0.89 mg/100 mL (crude oil) - 12.32 ± 0.43 mg/100 mL (degummed oil)	**1**	- No IC_50_ value mentioned - Breast and prostate cancer cells based on molecular docking	[29]
5.	*Kigelia pinnata* (Bignoniaceae family)	Fruits extracts	Significant cytotoxic effects against liver cancer (Huh-7), pancreatic cancer (PANC-1), colorectal cancer (Colo-205 and HT-29), gastric carcinoma (SNU-16), colorectal adenocarcinoma (SW620), and colon carcinoma (HCT116) cells. It has high free radical scavenging activity and iron ion reduction ability.	Not mentioned	-	- IC_50_ = 6.79 μg/mL, SW620 (colorectal adenocarcinoma) - IC_50_ = 8.69 μg/mL, SNU-16 (gastric carcinoma) - IC_50_ = 10.34 μg/mL, PANC-1 (pancreatic cancer) - IC_50_ = 65.55 μg/mL, Huh-7 (liver cancer) - IC_50_ = 52.92 μg/mL, Colo-205 (colorectal cancer) - IC_50_ = 91.32 μg/mL, HT-29 (colorectal cancer)	[33]
6.	*Haloxylon salicornicum* (Amaranthaceae family)	Whole of plant bodies extracts	Anticancer (liver cancer) activity through apoptosis (AO/EB staining) and cell cycle arrest (flow cytommetry)	Not mentioned	-	- Antioxidant IC_50_ = 4120 μg/mL - Liver cancer cells (HCAM)	[42]
7.	*Prunus persica* Linn. (Rosaceae family) & *Malpighia glabra* Linn. (Malpighiaceae family)	Leaves extracts	Anticancer activities on colon or HCT-116 and breast or MCF-7 cancer cells	α-tocopherol: 0.139 mg/100 g	**1**	- IC_50_ = *P*. *persica* cv. Desert red: 249.5 μg/mL, Florida prince: >1000 μg/mL, Swelling: 617 μg/mL, *M.glabra*: 490 μg/mL, breast cancer cells (MCF-7) - IC_50_ = *P. persica* cv. Desert red: 617 μg/mL, Florida prince: 617 μg/mL, Swelling: 490 μg/mL, *M. glabra*: 302 μg/mL - Colon cancer cells (HCT-116)	[43]
8.	*Petroselium crispum* L. & *Anethum graveolens* L. (Apiaceae family)	Shoot tissues extracts	Anticancer activities in HepG2 (carcinoma), Colo205 (colon carcinoma), 293 (embryonic kidney adenocarcinoma), and T24P (urinary bladder carcinoma) cell lines.	- *P. crispum* L. = 0.211 ± 0.09 μmole CO_2_/mole air, eCO_2_ - *A. graveolens* L. = 0.338 ± 0.06 μmole CO_2_/mole air, eCO_2_	**1**	- No IC_50_ value mentioned - MCF-7 (breast), HepG2 (liver), Colo205 (colorectal), and T24P (bladder) cancer cells	[44]
9.	*Cissus assamica* (Vitaceae family)	Stems purified extracts	Anticancer activity with cytotoxic effects on non-small cell lung carcinoma cells (NCI-H226) and colon cancer cells (HCT-116)	α-tocopherol: 200 mg tocopherol trimer IVa: 24.3 mg tocopherol trimer IVb: 28.1 mg 1,2-bis-(5- γ- tocopheril)- ethane:15.4 mg α-tocospiro B: 6.1 mg	α-tocopherol: **1** tocopherol trimer IVa: **7** tocopherol trimer IVb: **8** α-tocospiro B: **9**	Betulinic acid: - IC_50_ = 2.0 μM, non-small cell lung carcinoma cancer cells (NCI-H226) - IC_50_ = 1.6 μM, colorectal cancer cells (HCT-116) Epi-glut-5(6)- en-ol compound: - IC_50_ = 9.1 μM, non-small cell lung carcinoma cancer cells (NCI-H226) - IC_50_ = 6.0 μM, colorectal cancer cells (HCT-116)	[30]
10.	*Asclepias curassavica* L. (Apocynaceae family)	Whole of plant bodies extracts	Exhibits anticancer activities.	- vitamin E (tocopherol): 3.95% - γ -tocopherol: 0.79%	γ-tocopherol: **2**	No IC_50_ value and cancer cell specificity mentioned	[31]
11.	*Rubus fairholmianus* (Rosaceae family)	Root methanolic column subfraction (RFM) extract	Promotes apoptosis in MCF-7 cancer cells, characterized by decreased ATP, increased LDH, increased apoptosis, and Caspase 3/7 activity	Not mentioned	-	- No IC_50_ value mentioned - MCF-7 (breast) cancer cells	[45]
12.	*Michelia nilagirica* (Magnoliaceae family)	Bark extract	α -glucosidase inhibitory activity associated with blood sugar management as well as anticancer activity in HepG2 cells	2.43%	**1**	- IC_50_ = 303.26 ± 2.30 mg/mL - HepG2 cancer cells	[46]
13.	*Typhonium flagelliforme* Lodd. (Araceae family)	Shoots and tubers extracts	Not investigated	Vitamin E (α-tocopherol): 0.46%	**1**	Not investigated	[32]
14.	*Epilobium* Species (*E. hirsutum* L., *E. parviflorum* Schreb., *E. palustre* L., *E. dodonaei* Vill., and *E. angustifolium* L.) (Onagraceae family)	Aerial parts and leaves extracts	Anticancer activities on LNCap cell line through decreasing cellular viability.	- α-tocopherol: 9435.35 ± 398.51 ng/g d.w. - δ-tocopherol: 572.76 ± 33.14 ng/g d.w.	α-tocopherol: **1** δ-tocopherol: **3**	- *E. hirsutum* L. leaves (EtOH 50%), IC_50_ = 6.10 μg/mL - *E. hirsutum* L. leaves (EtOH 30%), IC_50_ = 6.69 μg/mL - *E. hirsutum* L. aerial parts (EtOH 30%), IC_50_ = 9.10 μg/mL - *E. parviflorum* aerial parts (EtOH 30%), IC_50_ = 8.49 μg/mL - *E. palustre* aerial parts (EtOH 30%), IC_50_ = 5.84 μg/mL - *E. dodonaei* aerial parts (EtOH 30%), IC_50_ = 6.85 μg/mL - *E. angustifolium* leaves (EtOH 30%), IC_50_ = 7.05 μg/mL - *E. angustifolium* aerial parts (EtOH 30%), IC_50_ = 10.56 μg/mL - LNCap cancer cells	[47]
15.	*Rubus* sp. (Rosaceae family)	Fruits purified extracts	Anticancer against HeLa cells with an increase in apoptosis reaching 67%	430 μg/g FW	**1**	- IC_50_ = 35 μg/mL - HeLa cells	[48]

Note: This table summarizes key findings from selected studies (Scopus 2016–2024) on tocopherol-containing herbal medicinal plants and their potential anticancer properties. Compounds marked (*) are semi-synthetic. Structure numbers in the “Structure Number” column refer to Figure 1.

Table 1 also include studies employing gas chromatography-mass spectrometry (GC-MS), liquid chromatographic-mass spectrometry (LC-MS), and high-performance liquid chromatography (HPLC) analysis to determine the amount/content of tocopherol, and using 3-(4,5-dimethylthiazol-2-yl)-2,5-diphenyltetrazolium bromide assay (MTT assay) to analyze the toxicity of test samples toward cancer cells.

In their study, Raletsena and Mongalo [28] identified 56 compounds in the crude acetone extract of *Bulbine anguistifolia* Poelln. Among these, the semi-synthetic derivative α-tocopheryl succinate was quantified at 4.16%. The study used gas chromatography time-of-flight mass spectrometry (GC-ToF-MS) analysis of the acetone extract to quantify the compound content. GC-ToF-MS is an analytical technique that combines the separation capabilities of gas chromatography (GC) with the susceptible and high-resolution detection of time-of-flight mass spectrometry (ToF-MS). Another case was with Kumar et al. [41], who used the GC-MS analysis method to obtain a more significant number of tocopherol variants. They managed to identify DL-α-Tocopherol as much as 14.75% in *Calligonum comosum* leaves. Many studies have used GC-MS analysis to measure high tocopherol content (Table 1).

Cortez-Trejo et al. [40] detailed the total content of tocols from the fruit of *Punica granatum* L. Tocols, including α-, δ-, γ-tocopherol, and β-, γ-tocotrienol, had a total amount of 32.1 ± 0.2 μg in white pomegranate, 69.4 ± 1.0 μg in pink pomegranate, and 110.0 ± 0.2 μg in red pomegranate. Red pomegranate has a higher nutrient and phytochemical content than pink and white pomegranate. *Moringa oleifera*, the seed part, has α-tocopherol content with α-tocopherol type of 13.76 ± 0.89 mg/100 mL in crude oil and 12.32 ± 0.43 mg/100 mL in oil that has undergone a degumming process [29]. *Prunus persica* Linn. from the Malpighiaceae family and *Malpighia glabra* Linn. from the Rosaceae family have high amounts of vitamin E (tocopherol) and vitamin C based on GC-MS analysis [43]. Vitamin E content (α-tocopherol) in *P. persica cv.* Florida prince reached 0.139 mg/100 g, and vitamin C in acerola (*M. glabra*) reached 34 mg/100 g.

As mentioned, most research in obtaining tocopherol variants is through GC-MS analysis. After knowing the variation of tocopherol content in various medicinal plant species through GC-MS analysis, it is essential to understand the factors that can affect the increase of this compound content. One method proven effective in increasing tocopherol content in plants is elevated CO_2_ (eCO_2_) treatment, as reported by Saleh et al. [44]. Elevated CO_2_ can stimulate metabolic pathways that produce secondary compounds, including antioxidants such as tocopherols. The eCO_2_ treatment can increase the accumulation of phenolic compounds and flavonoids, often associated with increased tocopherol content [44]. The total tocopherol content in *Petroselium crispum* L. before eCO_2_ treatment was 0.211 ± 0.09 μmole CO_2_/mole air, and after eCO_2_ treatment increased to 0.496 ± 0.02 μmole CO_2_/mole air, eCO_2_. The same treatment was also given to *Anethum graveolens* L. with total tocopherol content from 0.338 ± 0.06 μmole CO_2_/mole air to 0.421 ± 0.02 CO_2_/mole air, eCO_2_ [44]. The eCO_2_ treatment in the study could produce a significant increase in β- and γ-tocopherol levels in *P. crispum* yet not for *A. graveolens*, and δ-and α-tocopherol only increased for *A. graveolens*. However, total tocopherol levels increased in both plants. Other studies have also shown that increasing atmospheric CO_2_ concentrations can stimulate the production of (tocopherol) compounds in soybean (*Glycine max*) plants by increasing photosynthetic activity and protecting cell membranes from oxidative stress [49].

In addition to eCO_2_, other studies have explored various plant species for their unique tocopherol content and potential as bioactive agents. For example, Chan et al. [30] reported five types of tocopherols isolated from *Cissus assamica* species which showed significant potential in health studies. The five types of tocopherols had a total amount of tocopherols of 273.9 mg [30]. α-tocopherol was 200 mg, tocopherol trimer IVa was 24.3 mg, tocopherol trimer IVb was 28.1 mg, 1,2-bis-(5-γ-tocopheril)-ethane was 15.4 mg, and α-tocospiro B was 6.1 mg [42]. Among the five types of tocopherols, the first natural isolation of 1,2-bis-(5-γ-tocopheril)-ethane from *C. assamica* was identified. In addition, GC-MS analysis of *Asclepias curassavica* L. plants revealed that the vitamin E content, in the form of γ-tocopherol, reached 3.95% [31].

Furthermore, analytical techniques such as LC-MS and GC-MS not only help identify tocopherol content but also other compounds related to bioactive properties. In plants such as *Rubus fairholmianus* root methanolic column subfraction (RFM), α-tocopherol content and other chemicals lead to its biological function [45]. LC-MS analysis of RFM (at dominant LC-MS peaks at 469.71, 779, and 893) showed mass equivalents of α-tocopherol and flavonol glycosidic compounds. GC-MS analysis and Fourier transform infra-red (FT-IR) spectroscopy stated that 28 compounds were successfully detected from the extract of ethyl acetate of *Michelia nilagirica* stem bark, one of which was vitamin E (tocopherols) as much as 2.43% [46]. Likewise, the plant *Typhonium flagelliforme* Lodd. (family: Araceae) has an amount of vitamin E (α-tocopherol) as much as 0.46% in the tuber [32]. The α-tocopherol content is a new type of tocopherol detected in rodent tubers.

LC-MS analysis revealed that *Epilobium* species (*E. parviflorum* Schreb., *E. hirsutum* L., *E. dodonaei* Vill., *E. angustifolium* L., and *E. palustre* L.) contained α-tocopherol as much as 9435.35 ± 398.51 ng/g d.w and δ-tocopherol as much as 572.76 ± 33.14 ng/g d.w [47]. These amounts are the highest concentrations of α-tocopherol and δ-tocopherol. In addition to the species discussed, several other herbal medicinal plants exhibit high tocopherol content, although the total amount has not been specifically reported. These examples include *Kigelia pinnata* (fruit parts), *Haloxylon salicornicum* (throughout the plant), *Rubus* sp. (fruit parts), and *Typhonium flagelliforme* Lodd. (leaf and tuber parts), which are essential to explore further [32,42,48,50].

It is important to note that not all tocopherols have the same structure and function. Each type of tocopherol, such as α-, β-, γ-, and δ-tocopherol, has a unique role in plants and different anticancer activities [51]. In plants, α-tocopherol protects chloroplasts from damage caused by reactive oxygen species (ROS) during photosynthesis [52]. Meanwhile, β-tocopherol is less common in nature and is usually present in lower plant concentrations. γ-tocopherol plays a role in protecting plant cells from environmental stress, and δ-tocopherol is very effective in detoxifying harmful substances and protecting lipids in cell membranes from oxidation [53].

Apart from tocopherols, there are derived compounds such as tocopherol trimers IVa, IVb, 1,2-bis-(5-γ-tocopheril)-ethane, and α-tocospiro B, each with specific roles in responding to stress and maintaining cell membrane stability. Recent research has increasingly focused on understanding how these compounds are formed and accumulated under different environmental conditions, such as stress and elevated CO_2_ levels, to boost tocopherol content for health benefits and medical uses [54,55]. Tocopherols have beneficial biological effects by influencing gene expression, signal transmission, and altering cell function through protein-membrane interactions [51,56]. The variants of tocopherols in different medicinal plants can significantly impact their therapeutic properties, especially anticancer activity.

## 3. Herbal Medicinal Plants Containing Tocopherol with Anticancer Potential

Through a comprehensive understanding of tocopherol levels across a variety of plants and the factors that impact their biosynthesis, we can evaluate the potential therapeutic value of these compounds, particularly in the context of cancer treatment. Tocopherol have been shown to have potent anticancer activity (Table 1). All compounds presented in Table 1 are natural, except for those marked with an asterisk, which are semi-synthetic. For example, Raletsena and Mongalo [28] reported that the semi-synthetic tocopherol content of B. anguistifolia Poelln roots has anticancer potential against soybean lipoxygenase enzyme 15-LOX in addition to Cyclooxygenase-1 (COX-1) and COX-2. The acetone extract of this plant had a minimum inhibitory concentration (MIC) of 0.05 mg/mL for several pathogens. It showed antiproliferative effects against MCF-7 cells with LC_50_ concentrations of 25.33 and 22.46 μg/mL for acetone and dichloromethane extracts, respectively. The IC_50_ concentration of dichloromethane extract for 2,2′-Azino-bis(3-ethylbenzothiazoline-6-sulfonic acid) (ABTS) was 12.52 μg/mL, while the IC_50_ concentration of acetone extract against 2,2-Diphenyl-1-picrylhydrazyl (DPPH) was 2.88 μg/mL.

Kumar et al. [41] observed that the ethanol extract from *C. comosum* (EECC) demonstrates significant antiproliferative effects against MCF-7 (IC_50_ approximately 269 μg/mL) and MDA-MB-231 (IC_50_ approximately 258 μg/mL) breast cancer cells. Treatment with EECC induced notable apoptosis in both types of cancer cells, suggesting its potential as an effective anticancer agent. Additionally, Cortez-Trejo et al. [40] reported that pomegranate extract exhibits anticancer potential by inhibiting cancer cell growth. Moreover, the presence of tocopherol and phenolic compounds in pomegranate contributes to maintaining cell integrity, preventing genetic mutations, and reducing inflammation, which is a known risk factor for various cancer types [40]. Furthermore, molecular docking studies have shown that *M. oleifera* seed oil displays promising anticancer potential compared to degummed oil [29].

The methanolic concentrate of another species, the ripe fruit of *K. penata*, had significantly greater cytotoxic activities compared to the ethyl acetate extract and even compared to the drug doxorubicin, a commonly used chemotherapeutic agent [33]. Interestingly, the study showed that this extract exhibited substantial cytotoxic activity on numerous types of cancer cells, suchHuh-7 (liver cancer), PANC-1 (pancreatic cancer), Colo-205, HT-29, SW620, and HCT116 (all colorectal cancers), as well as SNU-16 (gastric carcinoma). Another study also showed that *P. crispum* L. and *A. graveolens* L. plants have anticancer potential in cell lines HepG2 (carcinoma), Colo205 (colon carcinoma), 293 (embryonic kidney adenocarcinoma), and T24P (urinary bladder carcinoma) [44]. This aligns with the results obtained from *M. glabra* plant extract, which showed anticancer activity against colon cancer cells with effectiveness equivalent to standard doxorubicin (0.1 μg/mL) [43]. These findings underscore the importance of further exploration of the *M. glabra* plant as a potential cancer drug.

However, not all plant extracts show similar anticancer activity. For example, *H. salicornicum* only has cytotoxic effects on liver cancer cells (HCAM), using microwave-assisted extraction (MAE) with an ethanol solvent [42]. The study showed significant toxicity activity at a dosage of 1000 μg/mL, with a cell death rate of 42.35%. This suggests that the anticancer activity of *H. salicornicum* may be more limited compared to other plants tested. On the other hand, tocopherols in *C. assamica* plants showed promising cytotoxicity potential against non-small cell lung carcinoma (NCI-H226) and colon cancer (HCT-116) with IC_50_ values in the range of 1.6 to 9.1 μM [30]. The significant decrease in cell viability at specific concentrations suggests that tocopherol can induce apoptosis, adding to the evidence that tocopherol plays a role in inhibiting cancer cell proliferation. For example, the plant *R. fairholmianus* showed a 67.73% decrease in cell viability at a concentration of 20 μg/mL, which induced *apoptosis* [45]. In addition, *Rubus* sp. has anticancer activity against HeLa cells, with an increase in apoptosis reaching 67% [48]. The PI3K/PTEN/AKT/mTOR pathway is a key signaling route involved in regulating cell growth, proliferation, and metabolism. Similar findings were also found in *M. nilagirica* and *Epilobium* [46,47].

Extraction of *T. flagelliforme* Lodd. has exhibited significant potential in mitigating breast cancer cell proliferation, attributed to its substantial tocopherol content [13,14,57]. Tocopherol, recognized for its potent anticancer properties, mitigates oxidative stress in cancer cells, thereby precipitating apoptosis and impeding cancer cell proliferation by safeguarding cellular membrane structures against oxidative harm [58]. These properties hold significance as oxidative stress contributes to the onset and progression of cancer. The foregoing studies affirm the antioxidative function of tocopherols in herbal medicinal plants, effectively curtailing cancer cell proliferation and instigating apoptosis.

Accordingly, these findings underscore the promising potential of plants containing tocopherol and methanolic extracts as prospective anticancer agents. Therefore, the utilization of tocopherol-containing plants as anticancer agents offers a potential natural approach to cancer therapy and could be the basis for developing plant-based anticancer drugs in the future.

## 4. Semi-Synthetic Tocopherols with Anticancer Potential

### 4.1. Semi-Synthetic Tocopherol Derivatives

Recent studies have significantly expanded our understanding of tocopherols and their derivatives in cancer research. A comprehensive review by Baj et al. [9] highlighted several promising semi-synthetic tocopherol derivatives. α-tocopherol succinate (α-TOS) has shown remarkable potential by inducing apoptosis in breast, prostate, and colon cancer cell lines through the disruption of mitochondrial function and generation of reactive oxygen species [9,59]. Another noteworthy derivative is tocopheryl polyethylene glycol succinate (TPGS), which enhances the bioavailability and efficacy of anticancer drugs. TPGS acts as a P-glycoprotein inhibitor, potentially overcoming multidrug resistance in cancer cells [9,60]. The development of redox-silent vitamin E analogs, such as α-tocopheryl ether-linked acetic acid (α-TEA) and α-tocopheroloxybutyric acid (α-TOS), represents another significant advancement. These compounds induce apoptosis independent of their antioxidant properties and have demonstrated effectiveness against breast, prostate, and ovarian cancer cells [9,61]. This research highlights the potential of modified tocopherols to enhance anticancer efficacy beyond the capabilities of their natural counterparts.

Synthetic derivatives of tocopherols have also shown promising results in cancer research. For instance, α-tocopherol succinate (α-TOS), a semi-synthetic derivative of α-tocopherol, has demonstrated potent anticancer effects across various cancer types. α-TOS has been shown to selectively induce apoptosis in malignant cells while sparing normal cells, making it an attractive candidate for cancer therapy [62,63]. Another synthetic analog, α-tocopherol phosphate (α-TP), has exhibited enhanced bioavailability and stability compared to natural tocopherols, potentially leading to improved anticancer efficacy [64]. These synthetic models provide valuable insights into structure-activity relationships and may guide the development of more potent tocopherol-based anticancer agents.

Moreover, tocopherols have shown potential as adjuvants in chemotherapy, enhancing the efficacy of conventional anticancer drugs while potentially reducing their side effects. For example, α-tocopherol has been found to increase the sensitivity of multidrug-resistant cancer cells to chemotherapeutic agents like doxorubicin [65]. Additionally, γ-tocopherol has demonstrated synergistic effects with cisplatin in lung cancer cells, enhancing the drug’s cytotoxicity while protecting normal cells from oxidative damage [66]. These findings suggest that combining tocopherols with standard chemotherapy regimens could improve treatment outcomes and reduce toxicity, opening new avenues for cancer treatment strategies.

### 4.2. Mechanisms of Action

Jiang et al. [67] elaborated on several mechanisms through which tocopherols exert their anticancer effects. A key mechanism involves the regulation of Peroxisome Proliferator-Activated Receptors (PPARs), particularly PPARγ, leading to decreased cell proliferation and increased apoptosis in various cancer types [67,68]. Additionally, tocopherols, especially γ-tocopherol, inhibit HMG-CoA reductase, which can lead to decreased cholesterol synthesis crucial for rapidly dividing cancer cells [67]. Beyond their antioxidant properties, tocopherols exhibit anticancer effects through various antioxidant-independent mechanisms. These include the inhibition of COX-2 and 5-LOX enzymes, reducing inflammation-related cancer progression [67,69], and the modulation of sphingolipid metabolism, which affects cell survival and death pathways [67]. Tocopherols also influence crucial cell signaling pathways such as NF-κB, STAT3, and MAPK, which play vital roles in regulating cell survival, proliferation, and apoptosis [67,70].

### 4.3. Tocopherols as Adjuvants in Chemotherapy

The potential of tocopherols as adjuvants in chemotherapy has been highlighted in several studies. Prasad et al. [71] demonstrated synergistic effects when γ-tocotrienol was combined with erlotinib in pancreatic cancer cells, while Pereira-Silva et al. [72] showed that α-tocopheryl succinate enhanced the efficacy of gemcitabine in pancreatic cancer models. Moreover, tocopherols have shown promise in reducing the side effects of chemotherapy. Vitamin E supplementation has been found to reduce cisplatin-induced cytotoxicity in cancer patients [73], and tocopherols have mitigated doxorubicin-induced cardiotoxicity in preclinical models [74]. This dual action of enhancing efficacy while reducing toxicity makes tocopherols particularly interesting as chemotherapy adjuvants.

### 4.4. Drug Delivery and Overcoming Resistance

In the realm of drug delivery, TPGS-based nanocarriers have improved the delivery and efficacy of paclitaxel in various cancer models [75]. D-α-tocopheryl polyethylene glycol 1000 succinate (TPGS) has enhanced the oral bioavailability of several anticancer drugs [60]. Furthermore, tocopherols and their derivatives have shown potential in overcoming drug resistance, a major challenge in cancer treatment. α-TOS has been observed to sensitize resistant leukemia cells to TRAIL-induced apoptosis [76], while TPGS inhibits P-glycoprotein, potentially reversing multidrug resistance in cancer cells [77,78]. These findings underscore the multifaceted potential of tocopherols and their derivatives in cancer treatment, both as standalone agents and as adjuvants to conventional therapies. The ongoing research in this field promises to open new avenues for more effective and less toxic cancer treatments, leveraging the benefits of both natural compounds and their synthetic modifications.

### 4.5. Future Perspective

The growing body of research on tocopherols and their derivatives in cancer treatment opens up several promising avenues for future investigation. One key area is the continued development of more potent semi-synthetic derivatives with enhanced anticancer properties. These could potentially combine the beneficial effects of natural tocopherols with improved bioavailability, targeting, or efficacy. For instance, recent work by Niculescu et al. [79] has shown promising results with a novel tocopherol-based nanoparticle that demonstrates enhanced tumor-targeting capabilities [79]. Future research could focus on optimizing these derivatives for specific cancer types or combining them with other anticancer agents for synergistic effects.

Additionally, further exploration of the molecular mechanisms underlying the anticancer effects of tocopherols is crucial. This could lead to the identification of new cellular targets or pathways that could be exploited for cancer therapy. Recent studies have begun to unravel the complex interplay between tocopherols and cancer cell metabolism. For example, Zhou et al. [80] discovered a novel interaction between α-tocopherol and the mTOR signaling pathway in breast cancer cells [80]. Expanding on these findings could potentially reveal new therapeutic targets.

Clinical trials represent another critical area for future research. While preclinical studies have shown promising results, more extensive clinical trials are needed to evaluate the efficacy and safety of tocopherols as adjuvants in various cancer types and treatment regimens. These trials could help establish optimal dosing strategies and identify specific cancer types that are most responsive to tocopherol-based interventions. A recent phase II trial by Kunnumakkara et al. [81] investigating the use of γ-tocotrienol in combination with gemcitabine for pancreatic cancer showed encouraging results [81]. Future large-scale, multi-center trials will be crucial in translating these findings into clinical practice.

Investigation of potential synergies between tocopherols and other natural compounds or conventional cancer therapies is another exciting area for future research. Combination therapies that leverage the unique properties of tocopherols could potentially lead to more effective treatment strategies with reduced side effects. For instance, Nesaretnam and Selvaduray [82] demonstrated a synergistic effect between δ-tocotrienol and curcumin in inhibiting prostate cancer growth in vitro and in vivo [82]. Exploring such combinations could open new avenues for cancer treatment.

Furthermore, the role of tocopherols in cancer prevention, particularly in high-risk populations, warrants further investigation. Long-term epidemiological studies and intervention trials are needed to establish the optimal intake of tocopherols for cancer prevention. A recent 10-year follow-up study by Donovan et al. [83] suggested that higher dietary intake of mixed tocopherols was associated with reduced risk of colorectal cancer in a Mediterranean population [83]. Expanding on such studies could help develop evidence-based prevention strategies.

Advancements in drug delivery systems, such as nanoformulations incorporating tocopherols, present opportunities for improving the targeted delivery of anticancer agents. This could enhance the efficacy of treatments while minimizing systemic toxicity. Recent work by Mehata et al. [75] and Gao et al. [84] on TPGS-based nanocarriers for the co-delivery of paclitaxel and siRNA has shown promising results in overcoming multidrug resistance in breast cancer [84]. Further research in this area could revolutionize cancer drug delivery.

As our understanding of the complex interactions between tocopherols and cancer biology deepens, we may uncover new paradigms in cancer therapy that could significantly impact patient outcomes. The multifaceted nature of tocopherols—from their antioxidant properties to their role in cell signaling and their potential as drug delivery vehicles—positions them as versatile tools in the fight against cancer. Future research directions should aim to leverage these diverse properties, potentially leading to novel, more effective, and less toxic approaches to cancer prevention and treatment.

## 5. Materials and Methods

This review explores the role of tocopherol-containing herbal medicinal plants as potential anticancer agents. We aim to identify current research trends, highlight promising plant sources of tocopherol for anticancer applications, and suggests a potential mechanisms underlying the anticancer properties of plant-derived tocopherols and semi-synthetic derivatives. To gather relevant literature, we conducted a comprehensive search of the Scopus database (https://scopus.com, accessed on 3 July 2024).

Scopus was chosen for its extensive coverage of peer-reviewed literature in the field of plant sciences and pharmacology. Scopus’s robust content curation process is overseen by the Content Selection and Advisory Board [37,38,39]. Our search strategy employed combinations of keywords including “tocopherol”, “medicinal plants”, “herbal plants”, and “bioactive anticancer compound” for tocopherol-containing plants. To specifically gather information on semi-synthetic tocopherols, we conducted an additional search using related terms, ensuring we included relevant studies on this topic.

We focused on original research articles published between 2016 and 2024, a period that reflects growing interest in the therapeutic potential of tocopherols in cancer research. Studies were selected based on their relevance to tocopherol-containing herbal medicinal plants and semi-synthetic tocopherols related to anticancer properties. We prioritized peer-reviewed articles that presented clear methodologies, sufficient data reporting, and specific results relating tocopherol to anticancer effects. From the collected literature, we extracted and analyzed information on plant species and parts used, biological activities, total tocopherol content, anticancer mechanisms and effects, as well as insights into semi-synthetic tocopherols. This approach allowed us to synthesize current knowledge and identify promising directions for future research in this field.

## 6. Conclusions

This review analyzed Scopus publications from 2016 to 2024 on tocopherol-containing plants and their anticancer potential. Fifteen herbal medicinal plants were identified as promising candidates, including *Bulbine anguistifolia* Poelln, *Punica granatum* L., *Moringa oleifera*, *Kigelia pinnata*, and *Typhonium flagelliforme* Lodd. The review explored tocopherols’ anticancer mechanisms, particularly apoptosis induction and cell cycle arrest. Key factors influencing the anticancer effects of tocopherols were examined, including their forms (α, β, γ, δ), concentrations, plant parts utilized, and their stability in various plants. The emerging research on semi-synthetic tocopherol derivatives highlighted their potential as adjuvants in chemotherapy, their role in enhancing drug delivery, and reducing side effects. This comprehensive analysis provides a foundation for future research in plant-based anticancer therapies, emphasizing the need for further investigation to develop novel, more effective, and less toxic cancer treatments. The findings contribute to a nuanced understanding of how tocopherol-containing plants can be leveraged in the development of future anticancer drugs. Future experimental studied, including in vitro and in vivo models, are essential to validate the anticancer potential of these tocopherol-rich plants and their derivatives.
